# Atomic-Layer Engineering of La_2−x_Sr_x_CuO_4_—La_2−x_Sr_x_ZnO_4_ Heterostructures

**DOI:** 10.3390/nano13152207

**Published:** 2023-07-29

**Authors:** Xiaotao Xu, Xi He, Anthony T. Bollinger, Xiaoyan Shi, Ivan Božović

**Affiliations:** 1 Condensed Matter Physics and Materials Science Division, Brookhaven National Laboratory, Upton, NY 11973, USA; 2 Department of Physics, The University of Texas at Dallas, Richardson, TX 75080, USA; 3 Department of Chemistry, Yale University, New Haven, CT 06520, USA; 4 Energy Sciences Institute, Yale University, West Haven, CT 06516, USA

**Keywords:** nanoengineering, superconductivity, cuprates, oxide molecular beam epitaxy

## Abstract

The fabrication of trilayer superconductor-insulator-superconductor (SIS) Josephson junctions with high-temperature superconductor (HTS) electrodes requires atomically perfect interfaces. Therefore, despite great interest and efforts, this remained a challenge for over three decades. Here, we report the discovery of a new family of metastable materials, La_2−x_Sr_x_ZnO_4_ (LSZO), synthesized by atomic-layer-by-layer molecular beam epitaxy (ALL-MBE). We show that LSZO is insulating and epitaxially compatible with an HTS compound, La_2−x_Sr_x_CuO_4_ (LSCO). Since the “parent” compound La_2_ZnO_4_ (LZO) is easier to grow, here we focus on this material as our insulating layer. Growing LZO at very low temperatures to reduce cation interdiffusion makes LSCO/LZO interfaces atomically sharp. We show that in LSCO/LZO/LSCO trilayers, the superconducting properties of the LSCO electrodes remain undiminished, unlike in previous attempts with insulator barriers made of other materials. This opens prospects to produce high-quality HTS tunnel junctions.

## 1. Introduction

Josephson junctions (JJs) are indispensable for superconducting electronics applications, including ultralow magnetic field and electromagnetic radiation sensors, ultrafast digital rapid single flux quantum circuits, and Josephson qubits, as well as for fundamental physics experiments, such as tunneling gap spectroscopy and study of quantum fluctuations of the vacuum [1,2,3,4,5,6,7,8]. Over the long history of JJs, many designs have been developed and explored [9,10,11,12,13,14,15,16], but in terms of device performance, reproducibility, and on-chip uniformity, the leading one remains the trilayer or “sandwich” JJ geometry.

To increase the operating temperature and the frequency range, much effort was invested in fabricating SIS trilayer junctions based on HTS cuprates [6,7,8,17,18,19,20,21,22,23,24,25,26,27,28,29,30,31,32,33,34,35]. A new problem encountered here was that in cuprates, the coherence length is very short, approximately 1–2 nm in-plane, and another order of magnitude shorter out-of-plane. Over the years, many different in-plane structures have been explored, such as grain boundary junctions, weak-link junctions, ramp-edge junctions, bicrystal junctions, etc. [19,20,22,23,24,25,26,27,28,29,30,33]. Some of these rely on deliberately created and controlled defects to produce a ‘weak link’. While one can obtain a decent JJ, or even make a good SQUID that way, the key hurdle on the path to real-life applications of such JJs in large-area superconducting digital circuits is that it is necessary but extremely difficult to achieve satisfactory on-chip uniformity, reproducibility, and yield. From half-a-century experience with low-*T_c_* superconductor electronics, the best candidates are trilayer ‘sandwich’ junctions, provided one can control the barrier thickness, properties, and integrity (i.e., ensure that there are no pinholes). In the case of cuprates, nature may be helping us, given that cuprates are naturally layered, quasi-two-dimensional (2D) materials. For this reason, their growth rate is much higher in-plane than out-of-plane, and they naturally form thin flakes; this fact favors *c*-axis-oriented thin films and heterostructures. The downside is that this requires the fabrication of SIS trilayers with ultrathin (less than 1 nm thick) insulator barriers, without pinholes, and with S–I interfaces that are sharp and perfect on the atomic scale.

The ALL-MBE technique has been producing the highest-quality cuprate thin films, complex heterostructures, and superlattices, with precisely engineered interfaces [7,8,36,37,38,39,40,41,42,43]. Using ALL-MBE, we can precisely control the layer thickness to one monolayer (ML) and have demonstrated that ultrathin barriers can be fabricated without any pinholes [7,8,34]. Just from the barrier height and thickness, one should expect a tunneling supercurrent in such *c*-axis JJ, without any defects. Nevertheless, the fabrication of SIS trilayer junctions has remained a formidable challenge [44,45,46,47,48,49]. A big part of the problem has been finding the perfect ‘I’ layer. The interfaces between cuprates and insulating oxides, commonly used as substrates such as SrTiO_3_, CaTiO_3_, BaTiO_3_, LaAlO_3_, etc., tend to be imperfect because these materials are electrostatically mismatched to cuprates. In SrTiO_3_, the atomic (SrO) and (TiO_2_) monolayers are charge-neutral; in contrast, LSCO has a layered-perovskite K_2_NiF_4_ or “214” structure in which (CuO_2_)^2-^ layers alternate with two (LaO)^+1^ layers. This causes a “polarization catastrophe” and the consequent structural reconstruction near the interface [44,48].

For this reason, the ideal insulating barrier material should have the same layered 214 structure, with a small divalent cation replacing Cu^2+^. The best results so far were achieved using the undoped La_2_CuO_4_ (LCO), the Mott-insulator parent compound, as the barrier material [7,8,34,36]. Growing LCO atop LSCO, or vice versa, is quasi-homoepitaxy, so the interface can be made atomically sharp, insofar that Sr interdiffusion can be suppressed. Using ALL-MBE, we synthesized and studied LSCO/LCO bilayers and LSCO/LCO/LSCO tunnel junctions extensively, and it was demonstrated that all LSCO layers remain superconducting. However, experiments showed that something prohibits the coherent supercurrent in LSCO/LCO/LSCO junctions. This has been known for two decades [34] and was reaffirmed in a more recent series of experiments [7,8]. Some theorists speculated that this may be due to the static or fluctuating antiferromagnetic order in the insulating LCO barrier, but a definitive microscopic understanding of this phenomenon is lacking. In any case, this is why we decided to search for different insulating barrier materials and specifically those that are not Mott insulators and prone to antiferromagnetic ordering.

Given that LaSrAlO_4_ (LSAO) is the preferred substrate for the growth of the highest-quality LSCO films, we experimented with LSAO barriers [49]. The problem we stumbled upon was the deterioration of superconducting properties of the LSCO electrodes, and the formation of “dead” LSCO layers near the interface. Even though we deposit only a 1 ML thick LSAO layer, the effective barrier is 5 ML thick [49], and indeed, in LSCO/LSAO/LSCO tunnel junctions, we observed no supercurrent either.

In this paper, we present two innovations, one in the synthesis protocol and another in the choice of the insulating barrier material, altogether significantly improving the heterostructure quality. First, studying the LSCO/LSAO/LSCO heterostructures, we developed a low-temperature growth technique to minimize the interdiffusion across the LSCO/LSAO interfaces. Next, we synthesized a new, metastable compound family, LSZO, verified that it is a good insulator, and demonstrated excellent heteroepitaxy with LSCO. Then we applied the low-temperature synthesis technique to grow LSCO/LZO/LSCO heterostructures by choosing LZO as the barrier (*x* = 0) and showed that the superconducting properties of LSCO remained undiminished. This opens the path to the fabrication of greatly improved HTS JJs soon.

## 2. Experiment: Discovery of Novel SIS

### 2.1. Growth of the Insulating LSAO Barrier at Low Temperature

One possible cause of the formation of dead LSCO layers near the LSAO–LSCO interface is the cation interdiffusion, predominantly Al to Cu intersite substitution. To address this issue, we introduced a growth protocol in which the substrate temperature (*T_s_*) is varied drastically for the superconducting (LSCO) and insulating (LSAO) layers. Generally, the higher *T_s_* is, the higher the surface mobility of deposited atoms, resulting in better film crystallinity and structural perfection. However, with increasing *T_s_*, bulk mobility increases as well, leading to enhanced cation intermixing across the interface in heterostructures. Therefore, we explored a path toward optimizing the tunnel junction properties, trying to strike a balance between these competing requirements. As illustrated next, the idea was to utilize, alternately, a higher *T_s_* to ensure excellent film crystallinity and a lower *T_s_* to improve interface sharpness.

**Figure 1:**
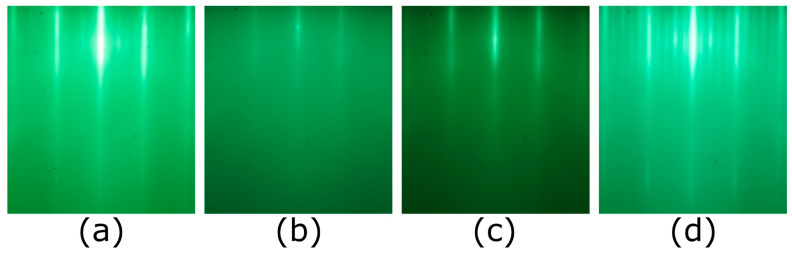
RHEED patterns of the sample surface during the growth of a [18 × LSCO + 1 × LSAO + 20 × LSCO] film. (**a**) RHEED image of the LSCO layer taken just before the growth of LSAO. The four sidebands between the main streaks are the prominent feature of an optimally doped LSCO film. (**b**) RHEED image taken after the LSAO layer was deposited at a low temperature, *T_s_* = 200 °C. (**c**) RHEED image of the same LSAO layer after it was heated up to *T_s_* = 630 °C. (**d**) RHEED image of the final LSCO layer grown on top of the LSAO layer.

We first synthesized 18 ML of LSCO at our typical growth temperature *T_s_* = 630 °C to serve as the bottom LSCO electrode. Then we cooled the film down and synthesized the 1 ML thick LSAO barrier layer at a very low temperature, *T_s_* = 200 °C. After that step was completed, we raised the temperature back up to 630 °C and verified that the reflection high-energy electron diffraction (RHEED) pattern recovered to that of the crystalline LSAO, proving that the constituent atoms of the LSAO layer recrystallized. Still, in this way, the intermixing between the LSAO layer and the LSCO layers underneath is significantly reduced, because now it largely relies on bulk interdiffusion. Then we grew another 20 ML thick LSCO slab to serve as the top superconducting electrode. During the entire growth process, the RHEED patterns of the sample surface (Figure 1a–d) showed no signs of secondary phase precipitation.

**Figure 2:**
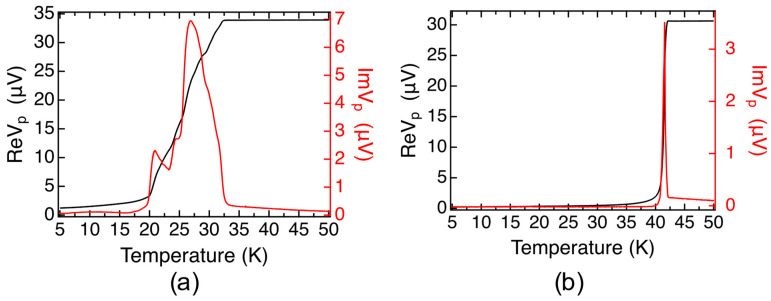
The comparison of MI data of the LSCO-LSAO-LSCO trilayer film and a single crystal LSCO film. Left ordinate: Re*V_p_*(*T*), the real (in-phase) component of *V_p_*. Right ordinate: Im*V_p_*(*T*), the imaginary part of *V_p_*. (**a**) MI data of a [18 × LSCO + 1 × LSAO + 20 × LSCO] trilayer film. (**b**) MI plot of an optimally doped LSCO film.

The mutual inductance (MI) measurement [50] on this SIS sample (Figure 2a) shows a gradual drop in the real part of emf (Re*V_p_*) and a broad peak in the imaginary part (Im*V_p_*), indicating a wide superconducting transition that starts at 33 K and ends at 20 K. In contrast, the MI plot of a typical single-phase, optimally doped LSCO film (Figure 2b) shows an extremely narrow superconducting transition in Im*V_p_*(*T*), with half-width-at-half-maximum (HWHM) of less than 0.5 K, indicating remarkable film quality and uniformity. The question is what causes this dramatic discrepancy.

**Figure 3:**
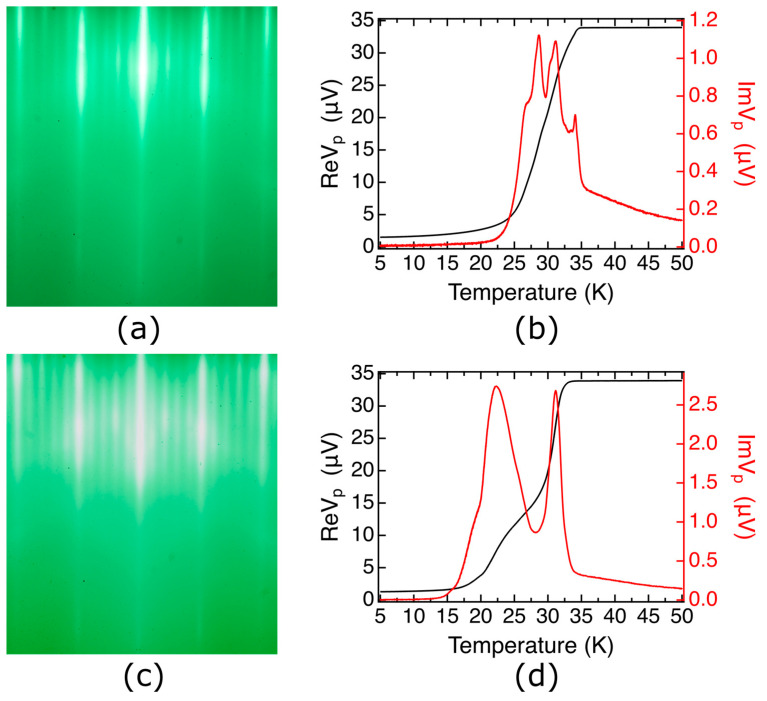
RHEED patterns and MI data of two [18 × LSCO + 1 × LSAO] films. (**a**,**c**) The RHEED patterns of the last LSCO layer of two films before the deposition of LSAO film. These RHEED patterns indicate that LSCO films underneath the LSAO layer are both atomically flat without defects. (**b**,**d**) Corresponding MI plots of two films after growth.

During the synthesis of the LSCO/LSAO/LSCO trilayers, we did not detect, by RHEED, the formation of any precipitates of unwanted phases, nor any indication of 3D growth [51]. Still, one could suspect that the wide superconducting transitions in such trilayers may originate in the top LSCO layer, e.g., because its crystallinity could be affected by the imperfect epitaxy atop the LSAO barrier layer grown at a low temperature. To test this hypothesis, we studied two [18 × LSCO + 1 × LSAO] heterostructures where LSCO was synthesized at *T_s_* = 630 °C and the LSAO layer at *T_s_* = 200 °C. The corresponding RHEED patterns and MI plots are shown in Figure 3. Neither of these films contains the top LSCO electrode layer, and yet both show broad superconducting transitions with multiple peaks resolved in Im*V_p_*(*T*) plots (Figure 3b,d). Since, in both heterostructures, the LSAO layer was grown at a low temperature and the film was not heated up subsequently, we can also rule out massive interdiffusion of Al and Cu atoms. The most plausible explanation is that due to the presence of Al atoms nearby, the underlying LSCO layers may lose some structural oxygen atoms, and thus the transition broadening originates from an inhomogeneous distribution of these oxygen vacancies. This is supported by our observation of gradual deterioration and the eventual disappearance of superconductivity in optimally doped LSCO films covered with Al, at room temperature. We observed the same effect with other metals that are reactive and prone to oxidation, such as Ca or Sr, as well.

For this reason, we turned to the exploration of new barrier layer materials.

### 2.2. La_2−x_Sr_x_ZnO_4_ Single-Crystal Films

As we pointed out above, to avoid the “polarization catastrophe”, the ideal barrier material should have the 214 structure, with a small divalent cation replacing Cu. Our earlier experiments with nickelates did not bring fruit, although epitaxy was reasonably good, so we looked elsewhere. It has been known in the HTS literature that the superconductivity in LSCO can be strongly suppressed by substituting a small amount (say 3%) of Cu with Zn. This led us to speculate that La_2−x_Sr_x_ZnO_4_ (LSZO), where 100% Cu is substituted with Zn, may turn out to be a good choice for insulating barriers in LSCO-based SIS tunnel junctions. However, LSZO has not been synthesized so far; it does not seem to be thermodynamically stable. Nevertheless, we tried to grow it as a metastable ‘artificial’ compound, using ALL-MBE and leveraging epitaxial stabilization and low-temperature, kinetically controlled synthesis.

Since LSZO is not a naturally occurring crystal, there is no reported information about its crystal structure and physical properties. Therefore, before growing the LSCO/LSZO heterostructures, we first studied the growth of single-crystal LSZO films. We performed synthesis experiments on a series of LSZO films with doping levels from *x* = 0 to *x* = 1.2 on LSAO substrates. The surface and structure of the films were monitored in real-time by RHEED and the surface morphology was characterized ex situ by atomic force microscopy (AFM). During the growth, the substrate temperature was maintained at *T_s_* = 650 °C. The LSZO films were synthesized in a layer-by-layer growth manner. To grow one LSZO monolayer, we begin by depositing two (La, Sr)O planes with a predetermined ratio of La and Sr atoms. After closing the La and Sr shutters, we open the Zn shutters and deposit one ZnO_2_ layer.

**Figure 4:**
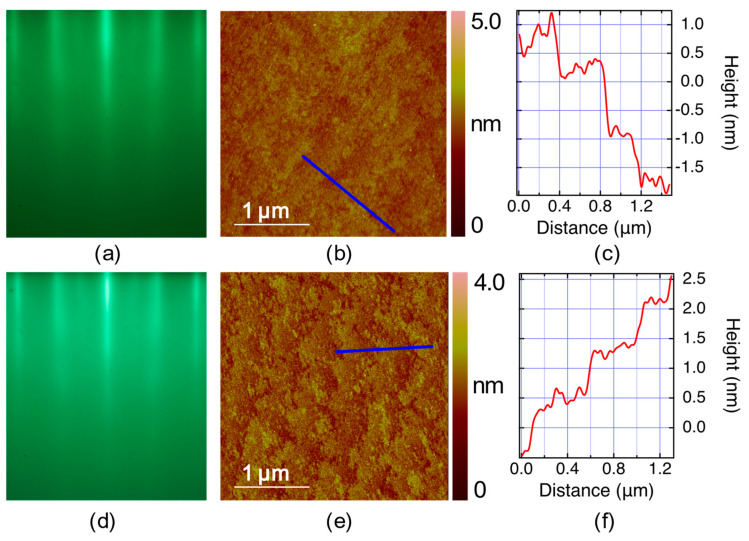
Characterization of the surface of LSZO films. (**a**) RHEED pattern of an LSZO (*x* = 0.3) film. Five main streaks originate from the (0, −2), (0, −1), (0, 0), (0, 1), and (0, 2) Bragg diffraction rods. (**b**) An AFM image of the surface of the same film as in (**a**). The field of view is 3 × 3 µm^2^. The RMS surface roughness is 0.16 nm. (**c**) The height profile along the blue line in (**b**) shows an atomically flat step-terrace structure. The height of one step corresponds to the height of one full unit cell of LSZO. (**d**) RHEED pattern of a LSZO (*x* = 0.2) film. (**e**) An AFM image of the film in (**d**). The RMS roughness is 0.25 nm. (**f**) The height profile along the blue line in (**e**) shows an atomically flat step-terrace structure.

Figure 4a,d show RHEED images of two LSZO films. The bright specular spot and long streaks indicate that the film crystallinity is high, and the surface is atomically flat. The five main streaks correspond to (0, −2), (0, −1), (0, 0), (0, 1), and (0, 2) Bragg diffraction rods. The separation of these streaks is the same as in the LSAO substrate, and since this distance is proportional to the inverse of the in-plane lattice constant, it indicates that these films are pseudomorphic with the substrate. A feature that is present in RHEED patterns of all LSZO films and absent in LSAO substrates is the multiple sidebands in between the main streaks.

The AFM images of the film surfaces (Figure 4b,e) show that the LSZO film follows the stepped terraces in the substrate. The RMS surface roughness in these two films is only 0.16 nm and 0.25 nm, respectively, indicating that the surfaces are atomically flat.

Thus, we have demonstrated that atomically smooth LSZO films can be epitaxially grown on the LSAO substrate using ALL-MBE, introducing LSZO as a new material family. The MI and transport measurements on LSZO films indicate that they are all insulating. In conclusion, LSZO emerged as a promising candidate material for insulating barriers in LSCO-based SIS tunnel junctions. In the rest of this paper, we focus on the *x* = 0 (“parent”) compound, La_2_ZnO_4_ (LZO), since it provided the best heteroepitaxy with LSCO.

### 2.3. Growth of the Insulating LZO Barrier at Low Temperature

**Figure 5:**
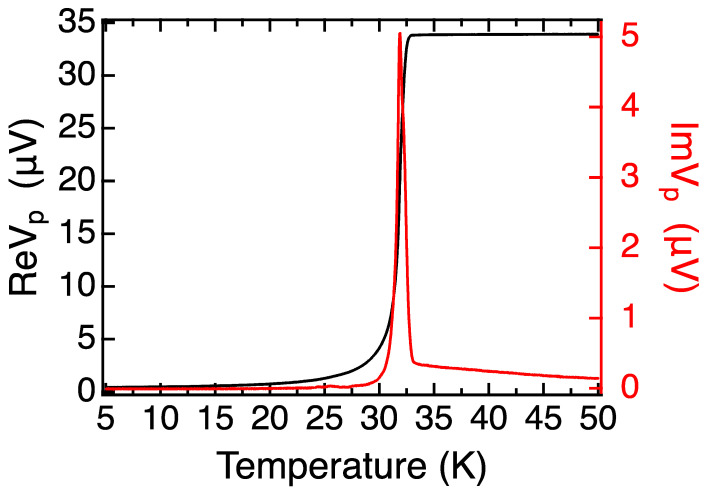
MI data of a [18 × LSCO + 1 × LZO] bilayer film.

Encouraged by this success, we explored the possibility of synthesizing LSCO/LZO heterostructures. We started by growing 18 ML of optimally doped LSCO on an LSAO substrate, at *T_s_* = 630 °C. Then we cooled the sample down to *T_s_* = 200 °C to deposit 1 ML of LZO, stopped the growth, and cooled the sample down to room temperature. The MI data of this [18 × LSCO + 1 × LZO] film (Figure 5) reveal a single narrow peak in Im*V_p_*(*T*), indicating a very sharp superconducting transition in this heterostructure. This is in stark contrast to the results obtained with [18 × LSCO + 1 × LSAO] films shown in Figure 2b,d, indicating that the LZO barrier grown at a low temperature has no detectable detrimental effect on the superconducting properties of the underlying LSCO film.

**Figure 6:**
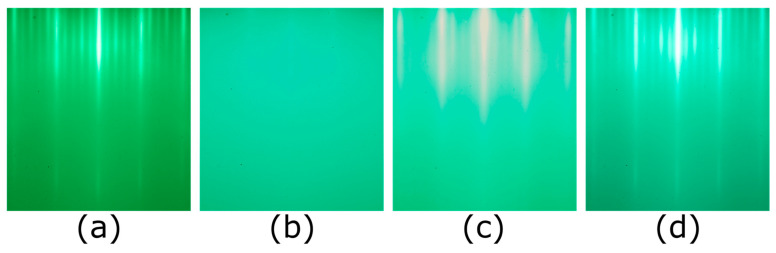
RHEED patterns of the sample surface during the growth of a [16 × LSCO + 1 × LZO + 10 × LSCO] film. (**a**) RHEED image of the LSCO layer taken just before the growth of LZO. The four sidebands between the main streaks are the prominent feature of an optimally doped LSCO film. (**b**) RHEED image taken after the LZO layer was deposited at a low temperature, *T_s_* = 200 °C. (**c**) RHEED image of the same LZO layer after it was heated up to *T_s_* = 630 °C. (**d**) RHEED image of the final LSCO layer grown on top of the LZO layer.

In the next set of experiments, we utilized the low-temperature growth technique to synthesize LSCO/LZO/LSCO trilayer films. In Figure 6, we illustrate the evolution of the RHEED patterns during the process of synthesis of a [16 × LSCO + 1 × LZO + 10 × LSCO] film. The pattern in Figure 6a was taken at the end of the growth of the bottom LSCO layers. In this image, a prominent feature is the presence of four sidebands, which implies a 5 × 1 or 5 × 5 surface reconstruction, which is characteristic of all LSCO films [42,43]. Figure 6b was taken after the sample was cooled down to *T_s_* = 200 °C and 1 ML of LZO was deposited at that temperature. The RHEED pattern nearly disappeared because this low surface temperature is insufficient for LZO crystallization. After that, we slowly increased the temperature back to *T_s_* = 630 °C, and the crystalline RHEED pattern reappeared (Figure 6c), indicating that the surface LZO layer underwent recrystallization. The RHEED features consist of long and sharp streaks and a bright specular spot, indicating epitaxial 2D growth without any 3D defects [51]. In contrast to the RHEED pattern of LSCO which features four sidebands, the LZO layer exhibits three sidebands. Although an atomistic understanding of the surface reconstruction is still missing when writing this paper, a different RHEED pattern indicates that the material is distinct from the underlying LSCO. When *T_s_* reached 630 °C, we started to grow the top LSCO layers. A transition from three sidebands to four sidebands occurred very fast, during the growth of the very first ML of LSCO on top of LZO. The abrupt transition between two different surface reconstructions once again implies that the LZO/LSCO interface is quite sharp. Subsequently, the same characteristic LSCO RHEED pattern persisted until the end of the growth process (Figure 6d), indicating excellent heteroepitaxy.

**Figure 7:**
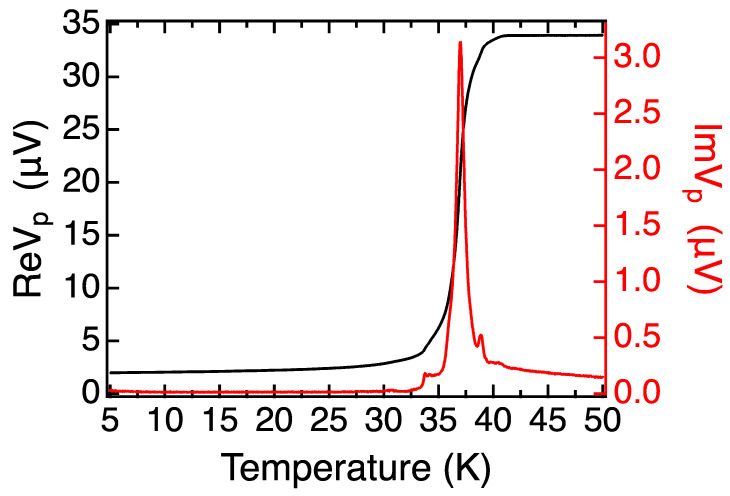
MI plot of the [16 × LSCO + 1 × LZO + 10 × LSCO] trilayer film.

The results of MI measurements on this LSCO/LZO/LSCO trilayer film are shown in Figure 7. The HWHM of the superconducting transition peak is less than 1 K. While we can see some small secondary peaks, this can still be considered as a sharp superconducting transition, indicating that the film is nearly homogeneous over the whole 10 × 10 mm^2^ area. The insertion of the LZO layer has no significant impact on the superconducting properties of the upper and lower LSCO layers. The SIS trilayers with LSCO electrodes and 1 ML LZO barriers thus present a significant improvement over previous ones with LSAO barriers, in terms of sharpness of the superconducting transition and homogeneity of the superfluid density.

## 3. Methods

The films reported here were grown in an ALL-MBE system that is specially designed for the growth of complex oxide superconducting materials [41,42]. It is equipped with 16 Knudsen effusion cells to provide stable atomic beam flux of multiple constituent elements and an ozone distillation system to supply pure ozone gas as the oxidizer. Several surface analytical tools, including a double-deflection RHEED and time-of-flight ion-scattering-and-recoil spectroscopy (TOF-ISARS), are integrated in situ to enable real-time monitoring of the morphology, crystal structure, and chemical composition of the surface during the growth.

In each of these synthesis experiments, RHEED was used in real-time, all the time. It has been well established that as long as any secondary-phase precipitates are nucleated on the surface, even if these are just a few tens of nanometers wide, they can be observed by RHEED. An atomically smooth surface generates a RHEED image that only contains streaks, similar to what is shown in Figure 1a–d, Figure 3a,c and Figure 6a,c,d. The better the film crystallinity, the longer and sharper the streaks are. Another positive sign is the appearance of the diagonal (“Kikuchi lines”) streaks that come from inelastic scattering. These features are also apparent in Figure 4a,d. In contrast, if secondary-phase precipitates are present on the surface, one also observes spots, originating from transmission through the precipitates. These spots are very broad and fuzzy if the precipitates are small (for example, 10–20 nm wide) and become sharper as the precipitates grow bigger. Since these phases (La_2_O_3_, SrO, and CuO) have different lattice constants from LSCO, they typically appear at different positions in the inverse space (i.e., in between the streaks).

Bragg diffraction spots can also be seen if there are no secondary-phase precipitates, but the surface of the single-phase (e.g., LSCO) film is rough, due to some island growth. The difference is that in this case, the extra spots sit right on the streaks that originate from the flat parts (since the lattice constant is the same in the islands and in the flat matrix under them). Again, no such modulation is seen in Figure 4a,d, so we are confident that no such islands exist on these surfaces.

After growth, every film is studied by AFM to provide further information about the film surface morphology. The above has been amply confirmed, with statistics in the thousands, by a posteriori AFM study of the films. When the RHEED images are of high quality such as in Figure 4a,d, the AFM images typically show RMS roughness less than 0.5 nm, which is less than the *c*-axis lattice constant (1.3 nm). Indeed, the AFM images in Figure 4b,e show RMS roughness of 0.16 and 0.25 nm, so these films are essentially flat without any detectable 3D defects or outgrowths.

Ex situ MI measurements are used to study the superconducting properties by detecting the temperature dependence of the diamagnetic response in films. The MI setup temperature is controlled with sub-millikelvin precision from room temperature down to *T* = 4 K. During the measurements, the drive coil operates at a fixed kHz frequency and creates a time-varying magnetic field that penetrates the thin film sample and induces alternating current (AC) in the pick-up coil at the other side, which is measured by a lock-in amplifier. When the film undergoes the superconducting transition, one would notice a drop in Re*V_p_*(*T*) in the pick-up coil showing the diamagnetic screening. At the same temperature, a peak arises in Im*V_p_*(*T*), which corresponds to the change in AC conductivity. The *T_c_* of the film can be read from the onset of the drop in Re*V_p_*(*T*) or the corresponding onset of the rise in Im*V_p_*(*T*). Moreover, because the whole setup is manufactured and calibrated to a high precision, given the geometry of coils and films, one can calculate the real number of the penetration depth λ and AC conductivity from the complex impedance by numerical inversion [50].

The MI technique is indeed very sensitive to the different superconducting transitions present in the measurement region. For example, if the film contains two regions with different critical temperatures, say *T_c1_* > *T_c2_*, upon cooling down, a superconducting transition occurs in the region with the higher *T_c1_*, and this triggers a change in the signal of the pick-up coil. The visually most apparent feature is a peak in the imaginary (dissipative) part Im*V_p_*(*T*), with the onset at *T_c1_*. As the temperature is lowered further to cause the other region to become superconducting, another separate peak in Im*V_p_*(*T*) will occur near *T_c2_*. This situation is illustrated well in Figure 2a and Figure 3b,d; some small secondary peaks are also seen in Figure 7. If inhomogeneity is not described by two or more discrete values of *T_c_* but rather by some distribution, the peaks in Im*V_p_*(*T*) will broaden, or even merge into a single broad peak.

As for the real (reactive) part, when a film becomes superconducting, it causes the value of the Re*V_p_*(*T*) to decrease because the magnetic field is screened. If there are regions with different transition temperatures, Re*V_p_*(*T*) will start dropping at *T_c1_* with some slope, and then at *T_c2,_* the slope will increase (i.e., there will be a kink).

So far, we were depicting a situation where the superconductivity in the film is inhomogeneous in the horizontal (in-plane, parallel to the substrate) direction. Similar effects can be observed if the sample is inhomogeneous vertically, i.e., if *T_c_* varies from layer to layer. If the film is thick enough, the field transmission through the layers with a higher *T_c_* may be very low, thus (almost) completely screening the signal from layers with a lower *T_c_*. However, this is not the case for films reported here, since they are all very thin.

To summarize this, although the coils are indeed macroscopic, any inhomogeneity in the *T_c_* distribution across the films can be detected by the MI measurements at high sensitivity. The multiple peaks in Im*V_p_*(*T*) are clear evidence of the inhomogeneity of the film. When only one peak is present, the HWHM of the peak is a good measure of the uniformity of the superfluid density. Qualitatively, a wider peak indicates that the variation in *T_c_* is larger, and homogeneity is worse. Quantitatively, if there are two peaks, at *T_c1_* > *T_c2_*, we can resolve them if *T_c1_*–*T_c2_* is larger than HWHM.

## 4. Discussion and Conclusions

Using ALL-MBE, we have synthesized a new family of compounds, LSZO, with *x* = 0 to *x* = 1.2. We demonstrated that LSZO is epitaxially quite compatible with LSAO and LSCO. We synthesized single-phase, atomically smooth LSZO films on LSAO substrates, as well as equally perfect LSCO/LZO bilayers and LSCO/LZO/LSCO trilayers. To reduce cation interdiffusion across the LSCO/LZO interfaces, we introduced a new ALL-MBE synthesis strategy—we deposited LSCO at a high temperature to ensure good crystallinity and sharp superconducting transition, then cooled the sample down to deposit LZO at a low temperature, preventing cation interdiffusion, ramped the temperature up gradually to recrystallize LZO, then grew the top LSCO electrode layer at a high temperature. In these heterostructures, LSCO maintains excellent superconducting properties equal to those of single-phase LSCO films.

Compared with a sharp transition of single-phase LSCO film in Figure 2b, the wide and multiple peaks seen in Figure 2a and Figure 3b,d imply that LSCO/LSAO/LSCO and LSCO/LSAO heterostructures have quite ‘bad’ transitions. Since these heterostructures are atomically flat, without obvious defects apparent in RHEED or AFM, we infer that the LSCO layers near the interface deteriorated due to the proximity to the LSAO layer, likely because of the electron charge transfer but perhaps also from some ionic displacements.

In stark contrast, the two different films in Figure 5 and Figure 7 show very sharp transitions. This implies that in LSCO/LZO (Figure 5), the LSCO layers underneath the LZO are quite homogeneous. Similarly, the MI data in Figure 7 indicate that in LSCO/LZO/LSCO heterostructures, the superconducting transitions of all LSCO layers, above and underneath the LZO, are very uniform. If in the top LSCO layers superconductivity were rendered bad by poor epitaxy with LZO, one would expect the MI plots to resemble those of LSCO/LSAO/LSCO and LSCO/LSAO heterostructures (Figure 2a and Figure 3b,d).

The MI data of Figure 5 and Figure 7 are taken from two different samples, and the values of *T_c_* differ by approximately 4 K. This is likely due to the lack of control during low-temperature growth; this is below the cut-off region of our current pyrometer, so we rely on keeping the heater power constant rather than using the PID control to stabilize the temperature. We are working on further hardware upgrades to improve this. Moreover, a remedy may exist in developing adequate post-growth annealing procedures that are different from those we optimized for the synthesis of single-crystal thin films that are grown at high and constant temperatures. However, note that these sample-to-sample *T_c_* variations are not critical to our objective in this paper, which is the discovery of a new metastable compound (LZO) that shows superior performance as an insulator barrier in LSCO-based SIS heterostructures layers. Notably, LZO does not cause deterioration of superconducting properties of the nearest LSCO layers, so in this way, we have solved the most critical and long-standing problem in the fabrication of HTS tunnel junctions.

In summary, we have shown that LZO is a very promising candidate for the insulator barrier material in HTS heterostructures, including SIS tunnel junctions, which we plan to fabricate and study in the near future.

## Data Availability

All data are available upon reasonable request.

## References

[1] Barone A., Paternò G. (1982). Physics and Applications of the Josephson Effect.

[2] Likharev K.K., Lukens J. (1986). Dynamics of Josephson Junctions and Circuits.

[3] Kulik I.O., Yanson I.K. (1972). Josephson Effect in Superconducting Tunnel Structures.

[4] Van Duzer T., Turner C.W. (1981). Principles of Superconductive Devices and Circuits.

[5] Wolf E.L., Arnold G.B., Gurvitch M.A., Zasadzinski J.F. (2017). Josephson Junctions: History, Devices, and Applications.

[6] Tafuri F. (2019). Fundamentals and Frontiers of the Josephson Effect.

[7] Zhou P., Chen L., Sochnikov I., Wu T.C., Foster M.S., Bollinger A.T., He X., Božović I., Natelson D. (2020). Tunneling Spectroscopy of C-Axis Epitaxial Cuprate Junctions. Phys. Rev. B.

[8] Zhou P., Chen L., Liu Y., Sochnikov I., Bollinger A.T., Han M.G., Zhu Y., He X., Božović I., Natelson D. (2019). Electron Pairing in the Pseudogap State Revealed by Shot Noise in Copper Oxide Junctions. Nature.

[9] Caldeira A.O., Leggett A.J. (1981). Influence of Dissipation on Quantum Tunneling in Macroscopic Systems. Phys. Rev. Lett..

[10] Devoret M.H., Martinis J.M., Clarke J. (1985). Measurements of Macroscopic Quantum Tunneling out of the Zero-Voltage State of a Current-Biased Josephson Junction. Phys. Rev. Lett..

[11] Martinis J.M., Devoret M.H., Clarke J. (1987). Experimental Tests for the Quantum Behavior of a Macroscopic Degree of Freedom: The Phase Difference across a Josephson Junction. Phys. Rev. B.

[12] Caldeira A.O., Leggett A.J. (1983). Quantum Tunnelling in a Dissipative System. Ann. Phys. (N. Y.).

[13] Clarke J., Wilhelm F.K. (2008). Superconducting Quantum Bits. Nature.

[14] Martinis J.M. (2009). Superconducting Phase Qubits. Quantum. Inf. Process..

[15] McDermott R. (2009). Materials Origins of Decoherence in Superconducting Qubits. IEEE Trans. Appl. Supercond..

[16] Devoret M.H., Schoelkopf R.J. (2013). Superconducting Circuits for Quantum Information: An Outlook. Science (1979).

[17] Schrieffer J.R., Brooks J.S., Schrieffer J.R., Brooks J.S. (2007). Handbook of High-Temperature Superconductivity.

[18] Tafuri F., Massarotti D., Galletti L., Stornaiuolo D., Montemurro D., Longobardi L., Lucignano P., Rotoli G., Pepe G.P., Tagliacozzo A. (2013). Recent Achievements on the Physics of High-T_C_ Superconductor Josephson Junctions: Background, Perspectives and Inspiration. J. Supercond. Nov. Magn..

[19] Hilgenkamp H., Mannhart J. (2002). Grain Boundaries in High-T_c_ Superconductors. Rev. Mod. Phys..

[20] Tafuri F., Kirtley J.R. (2005). Weak Links in High Critical Temperature Superconductors. Rep. Prog. Phys..

[21] Tsuei C.C., Kirtley J.R. (2000). Pairing Symmetry in Cuprate Superconductors. Rev. Mod. Phys..

[22] Chaudhari P., Mannhart J., Dimos D., Tsuei C.C., Chi J., Oprysko M.M., Scheuermann M. (1988). Direct Measurement of the Superconducting Properties of Single Grain Boundaries in Y_1_Ba_2_Cu_3_O_7_. Phys. Rev. Lett..

[23] Tafuri F., Miletto Granozio F., Carillo F., Di Chiara A., Verbist K., Van Tendeloo G. (1999). Microstructure and Josephson Phenomenology in 45° Tilt and Twist YBa_2_Cu_3_O_7-δ_ Artificial Grain Boundaries. Phys. Rev. B Condens. Matter. Mater. Phys..

[24] Lombardi F., Tafuri F., Ricci F., Granozio F.M., Barone A., Testa G., Sarnelli E., Kirtley J.R., Tsuei C.C. (2002). Intrinsic D-Wave Effects in YBa_2_Cu_3_O_7-δ_ Grain Boundary Josephson Junctions. Phys. Rev. Lett..

[25] Longobardi L., Massarotti D., Stornaiuolo D., Galletti L., Rotoli G., Lombardi F., Tafuri F. (2012). Direct Transition from Quantum Escape to a Phase Diffusion Regime in YBaCuO Biepitaxial Josephson Junctions. Phys. Rev. Lett..

[26] Bauch T., Lombardi F., Tafuri F., Barone A., Rotoli G., Delsing P., Claeson T. (2005). Macroscopic Quantum Tunneling in D-Wave YBa_2_Cu_3_O_7-δ_ Josephson Junctions. Phys. Rev. Lett..

[27] Bauch T., Lindström T., Tafuri F., Rotoli G., Delsing P., Claeson T., Lombardi F. (2006). Quantum Dynamics of a D-Wave Josephson Junction. Science (1979).

[28] Mannhart J., Chaudhari P., Dimos D., Tsuei C.C., McGuire T.R. (1988). Critical Currents in [001] Grains and across Their Tilt Boundaries in YBa_2_Cu_3_O_7_ Films. Phys. Rev. Lett..

[29] Herrmann K., Kunkel G., Siegel M., Schubert J., Zander W., Braginski A.I., Jia C.L., Kabius B., Urban K. (1995). Correlation of YBa_2_Cu_3_O_7_ Step-edge Junction Characteristics with Microstructure. J. Appl. Phys..

[30] Poppe U., Divin Y.Y., Faley M.I., Wu J.S., Jia C.L., Shadrin P., Urban K. (2001). Properties of YBa_2_Cu_3_O_7_ Thin Films Deposited on Substrates and Bicrystals with Vicinal Offcut and Realization of High IcRn Junctions. IEEE Trans. Appl. Supercond..

[31] Ozyuzer L., Zasadzinski J.F., Kendziora C., Gray K.E. (2000). Quasiparticle and Josephson Tunneling of Overdoped Bi_2_Sr_2_CaCu_2_O_8+δ_ Single Crystals. Phys. Rev. B.

[32] Sharoni A., Koren G., Millo O. (2001). Correlation of Tunneling Spectra with Surface Nanomorphology and Doping in Thin YBa_2_Cu_3_O_7-δ_ Films. Europhys. Lett..

[33] Smilde H.J.H., Blank D.H.A., Gerritsma G.J., Hilgenkamp H., Rogalla H. (2002). D-Wave–Induced Josephson Current Counterflow in YBa_2_Cu_3_O_7_/NB Zigzag Junctions. Phys. Rev. Lett..

[34] Bozovic I., Logvenov G., Verhoeven M.A.J., Caputo P., Goldobin E., Geballe T.H. (2003). No Mixing of Superconductivity and Antiferromagnetism in a High-Temperature Superconductor. Nature.

[35] Shim H., Chaudhari P., Logvenov G., Bozovic I. (2008). Electron-Phonon Interactions in Superconducting La_1.84_Sr_0.16_CuO_4_ Films. Phys. Rev. Lett..

[36] Bollinger A.T., He X., Caruso R., Xu X., Shi X., Božović I. (2023). Methods to Create Novel La_2-x_Sr_x_CuO_4_ Devices with Multiple Atomically Sharp Interfaces. Condens. Matter..

[37] Eckstein J.N., Bozovic I. (1995). High-Temperature Superconducting Multilayers and Heterostructures Grown by Atomic Layer-By-Layer Molecular Beam Epitaxy. Annu. Rev. Mater. Sci..

[38] Schlom D.G., Harris J.S., Farrow R.F.C. (1995). MBE Growth of High T_c_ Superconductors. Molecular Beam Epitaxy.

[39] Naito M., Sato H. (1995). Stoichiometry Control of Atomic Beam Fluxes by Precipitated Impurity Phase Detection in Growth of (Pr,Ce)_2_CuO_4_ and (La,Sr)_2_CuO_4_ Films. Appl. Phys. Lett..

[40] Naito M., Sato H., Yamamoto H. (1997). MBE Growth of (La,Sr)_2_CuO_4_ and (Nd,Ce)_2_CuO_4_ Thin Films. Phys. C Supercond. Its Appl..

[41] Bozovic I. (2001). Atomic-Layer Engineering of Superconducting Oxides: Yesterday, Today, Tomorrow. IEEE Trans. Appl. Supercond..

[42] Xu X., He X., Shi X., Božović I. (2022). Synthesis of La_2−x_Sr_x_CuO_4_ Films via Atomic Layer-by-Layer Molecular Beam Epitaxy. APL Mater..

[43] He X., Xu X., Shi X., Božović I. (2023). Optimization of La_2-x_Sr_x_CuO_4_ Single Crystal Film Growth via Molecular Beam Epitaxy. Condens. Matter..

[44] Bozovic I., Eckstein J.N. (1996). Superconductivity in Cuprate Superlattices. Physical Properties of High Temperature Superconductors V.

[45] Naito M., Yamamoto H., Sato H. (2000). Intrinsic Problem of Cuprate Surface and Interface: Why Good Tunnel Junctions Are Difficult to Fabricate. Phys. C Supercond..

[46] Nakagawa N., Hwang H.Y., Muller D.A. (2006). Why Some Interfaces Cannot Be Sharp. Nat. Mater..

[47] He J., Klie R.F., Logvenov G., Bozovic I., Zhu Y. (2007). Microstructure and Possible Strain Relaxation Mechanisms of La_2_CuO_4+δ_ Thin Films Grown on LaSrAlO_4_ and SrTiO_3_ Substrates. J. Appl. Phys..

[48] Pentcheva R., Pickett W.E. (2009). Avoiding the Polarization Catastrophe in LaAlO_3_ Overlayers on SrTiO_3_(001) through Polar Distortion. Phys. Rev. Lett..

[49] Xu X., He X., Bollinger A.T., Han M., Zhu Y., Shi X., Božović I. (2023). A Method to Probe the Interfaces in La_2-x_Sr_x_CuO_4_-LaSrAlO_4_-La_2−x_Sr_x_CuO_4_ Trilayer Junctions. Condens. Matter..

[50] He X., Gozar A., Sundling R., Božović I. (2016). High-Precision Measurement of Magnetic Penetration Depth in Superconducting Films. Rev. Sci. Instrum..

[51] Bozovic I., Eckstein J.N. (1995). Analysis of Growing Films of Complex Oxides by RHEED. MRS Bull..

